# Online HPLC‐Guided Screening of Chemical Composition and Bioactivity in *Capparis ovata* subsp. *canescens*

**DOI:** 10.1002/cbdv.71625

**Published:** 2026-08-25

**Authors:** Gokhan Zengin, Shakeel Ahmed, Ismail Senkardes, Milena Terzic, Ozan Emre Eyupoglu, Sakina Yagi

**Affiliations:** ^1^ Department of Biology, Science Faculty Selcuk University Konya Türkiye; ^2^ Department of Pharmaceutical Botany Pharmacy Faculty Marmara University Istanbul Türkiye; ^3^ Faculty of Technology Novi Sad University of Novi Sad Novi Sad Serbia; ^4^ School of Pharmacy Department of Biochemistry Istanbul Medipol University Istanbul Türkiye; ^5^ Department of Biology University of Khartoum Khartoum Sudan

**Keywords:** antioxidants, bioactive compounds, *C. ovata*, enzyme inhibition, online HPLC

## Abstract

The present study evaluated the biological activities and online HPLC‐DAD profiles of five extracts of *Capparis ovata* subsp. *canescens* prepared with ethyl acetate (EA), dichloromethane (DCM), ethanol, 70% ethanol/water, and water. Online HPLC‐DAD with post‐column antioxidant detection was used to profile tentatively annotated antioxidant‐responsive metabolites at 280, 595, 517, 734, and 450 nm. Antioxidant activity was assessed using DPPH, ABTS, FRAP, CUPRAC, phosphomolybdenum (PBD), and metal‐chelating assays. Eight antioxidant‐responsive markers—catechin, cinnamic acid, caffeic acid, γ‐tocopherol, apigenin, quercetin‐3‐O‐hexoside, myristoleic acid, and kaempferol‐3‐O‐rutinoside—were tentatively annotated from their chromatographic behavior, UV‐DAD characteristics, and post‐column antioxidant responses. Ethanol and ethanol/water extracts showed the strongest DPPH (82.35–82.97 mg TE/g), ABTS (93.68–122.00 mg TE/g), FRAP (98.46–116.84 mg TE/g), and CUPRAC (155.43–205.02 mg TE/g) activities. In the PBD assay, EA, ethanol, and DCM showed the highest activities (2.11–2.39 mmol TE/g), with EA displaying the highest numerical value (2.39 ± 0.10 mmol TE/g). These findings support further investigation of *C. ovata* as a source of antioxidant and enzyme‐inhibitory constituents, while structural confirmation and dose‐response studies remain necessary.

## Graphical abstract

Solvent polarity modulates metabolite recovery and bioactivities in *Capparis ovata* extracts.

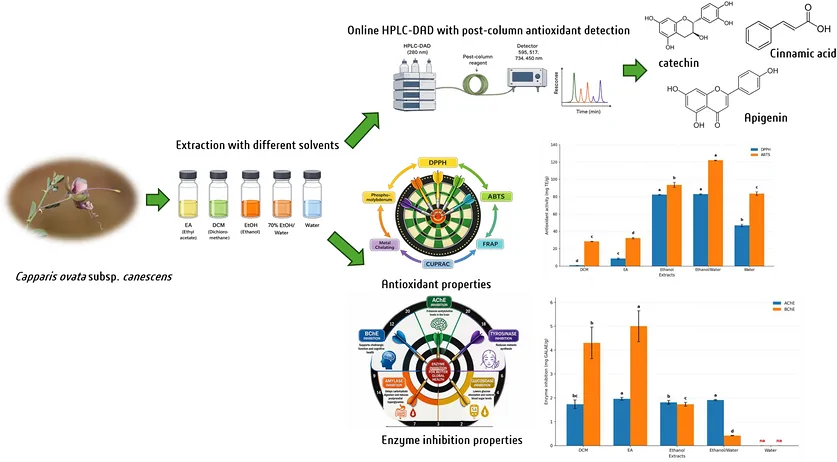

## 1 Introduction

Phytochemicals are plant‐derived, non‐nutritive compounds that are widely investigated for health‐promoting applications [1, 2, 3]. The caper *Capparis ovata* Desf. (Capparaceae) is distributed throughout the Mediterranean region and is an economically important plant with traditional uses for rheumatism, headache, and toothache [3, 4, 5].

Caper species have been reported to possess antioxidant, hypolipidemic, anti‐inflammatory, and hepatoprotective activities. Their fruits contain phenolic compounds, flavonoids, tocopherols, vitamin C, and carotenoids that may contribute to antioxidant effects [6, 7, 8]. *C. ovata* grows widely as a wild species in Turkey, is consumed as food, and has a long history of use in traditional medicine [3, 5, 8, 9].

Beyond its traditional uses, *C. ovata* has demonstrated several pharmacologically relevant effects [9, 10]. Evidence from published studies suggests that the methanolic extract of *C. ovata* fruits possesses antinociceptive activity [11]. Gazioglu et al. also reported moderate‐to‐strong anti‐inflammatory activity for triterpenoids and steroids isolated from *C. ovata* [3]. Collectively, these findings support continued investigation of caper‐derived constituents for functional‐food and nutraceutical applications [5, 6, 9, 11, 12, 13].

Earlier studies have examined the extraction of phenolic compounds from caper fruits using solvents of different polarities, including water, methanol, ethyl acetate (EA), and ethanol/water mixtures [6, 14]. Because plant phytochemicals differ widely in polarity and structure, no single solvent system can efficiently recover the full range of target compounds [15, 16]. Alcoholic solvents, such as ethanol and methanol, are widely used to extract phenolic compounds because of their broad solubilizing capacity [7, 17, 18, 19, 20]. Nevertheless, solvent selection must also consider toxicity, biodegradability, flammability, cost, and suitability for the intended application [21, 22, 23, 24].

Despite previous studies on the phytochemical and biological properties of *C. ovata*, the influence of solvent polarity on antioxidant and enzyme‐inhibitory activities, and on the antioxidant‐responsive chromatographic profile, remains insufficiently characterized. Therefore, this study compared five extracts prepared with solvents of different polarities, determined their total phenolic and flavonoid contents (TPC and TFC), assessed their antioxidant and enzyme‐inhibitory activities, and used online HPLC‐DAD post‐column assays to prioritize major redox‐active chromatographic features. To the best of our knowledge, this is the first study to combine online HPLC‐DAD post‐column antioxidant profiling with a systematic polarity‐based comparison of five *C. ovata* subsp. *canescens* extracts, thereby linking tentatively annotated metabolites with their antioxidant‐response profiles and extending previous studies that primarily reported bulk activity.

## 2 Materials and Methods

### 2.1 Plant Collection

Plant material was collected in 2022 from Küçükkuyu, Ayvacık, Çanakkale, in the Aegean region of Türkiye. Dr. Ismail Senkardes performed the botanical identification, and a voucher specimen was deposited in the Herbarium of the Faculty of Pharmacy, Marmara University (voucher no. MARE 22878). The aerial parts were separated after collection, air‐dried at room temperature in a shaded and well‐ventilated area, and milled to a fine powder. The powdered material was stored in sealed opaque containers under stable conditions until extraction.

### 2.2 Plant Extract Preparation

Four organic solvent systems—dichloromethane (DCM), EA, ethanol, and 70% (v/v) ethanol/water—were used for maceration. For each organic extraction, 10 g of powdered plant material was mixed with 200 mL of solvent and maintained at room temperature for 24 h. The aqueous extract was prepared separately by infusing 10 g of plant material in 200 mL of hot distilled water for 15 min. Organic solvents were removed under reduced pressure using a rotary evaporator, whereas the water extract was freeze‐dried. The five extracts analyzed throughout the study were therefore DCM, EA, ethanol, 70% ethanol/water, and water; methanol was not used as an extraction solvent. Each extraction was performed independently three times, yielding three independently prepared extracts for each solvent system (*n* = 3).

### 2.3 TPC and TFC

TPC and TFC were determined using validated colorimetric procedures [25]. Gallic acid and rutin calibration curves were used, and the results were expressed as milligrams of gallic acid equivalents per gram of dried extract (mg GAE/g) and milligrams of rutin equivalents per gram of dried extract (mg RE/g), respectively (Table 1).

**TABLE 1:** Total phenolic and flavonoid contents of the tested extracts.

Extracts	TPC (mg GAE/g)	TFC (mg RE/g)
DCM	19.89 ± 0.28^d^	1.81 ± 0.35^d^
EA	21.80 ± 0.32^c^	1.91 ± 0.30^d^
Ethanol	40.95 ± 0.44^a^	11.39 ± 0.77^c^
Ethanol/Water	28.33 ± 0.30^b^	53.58 ± 0.41^a^
Water	15.63 ± 0.05^e^	16.61 ± 0.05^b^

*Note*: Values are presented as mean ± SD from three independently prepared extracts for each solvent system (*n* = 3). One‐way ANOVA followed by Tukey's post hoc test was used. Within each column, different superscript letters indicate significant differences among extracts (*p* < 0.05). Abbreviations: DCM, dichloromethane; EA, ethyl acetate; GAE, gallic acid equivalents; RE, rutin equivalents.

### 2.4 Antioxidant Assays

Antioxidant activity was determined using DPPH, ABTS, CUPRAC, FRAP, phosphomolybdenum (PBD), and ferrous‐ion‐chelating assays according to the laboratory protocol based on ref. [26]. For the DPPH assay, 1.0 mL of extract solution was mixed with 4.0 mL of a 0.004% (w/v) DPPH solution in methanol, incubated for 30 min at room temperature in the dark, and the absorbance was measured at 517 nm. The ABTS radical cation was generated from 7 mM ABTS and 2.45 mM potassium persulfate, kept in the dark at room temperature, and diluted to an absorbance of 0.700 ± 0.020 at 734 nm; 1.0 mL of extract solution was then mixed with 2.0 mL of the working solution and measured at 734 nm after 30 min. For CUPRAC, 0.5 mL of extract solution was combined with 1.0 mL each of 10 mM CuCl_2_, 7.5 mM neocuproine, and 1 M ammonium acetate buffer (pH 7.0), incubated for 30 min at room temperature, and read at 450 nm. For FRAP, 0.1 mL of extract solution was mixed with 2.0 mL of freshly prepared reagent containing 0.3 M acetate buffer (pH 3.6), 10 mM TPTZ in 40 mM HCl, and 20 mM FeCl_3_ in a 10:1:1 (v/v/v) ratio; absorbance was measured at 593 nm after 30 min. For the PBD assay, 0.3 mL of extract solution was combined with 3.0 mL of reagent containing 0.6 M H_2_SO_4_, 28 mM sodium phosphate, and 4 mM ammonium molybdate, incubated at 95°C for 90 min, and read at 695 nm. Metal‐chelating activity was measured by adding 2.0 mL of extract solution to 0.05 mL of 2 mM FeCl_2_, initiating the reaction with 0.2 mL of 5 mM ferrozine, and recording absorbance at 562 nm after 10 min at room temperature. Appropriate reagent blanks were included. DPPH, ABTS, CUPRAC, and FRAP results were expressed as mg TE/g; PBD activity as mmol TE/g; and metal‐chelating activity as mg EDTAE/g. For each solvent system, antioxidant assays were performed on three independently prepared extracts, and the results are presented as mean ± SD (*n* = 3).

### 2.5 Enzyme Inhibitory Activity Assays

Enzyme‐inhibitory activities were determined according to the laboratory protocol based on ref. [26]. For AChE and BChE inhibition, 50 µL of extract solution, 125 µL of 3 mM DTNB, and 25 µL of enzyme solution in Tris‐HCl buffer (pH 8.0; AChE, 0.265 U/mL, or BChE, 0.026 U/mL) were incubated for 15 min at 25°C. The reaction was initiated with 25 µL of 15 mM acetylthiocholine iodide or 1.5 mM butyrylthiocholine chloride, and absorbance was recorded at 405 nm after 15 min; galantamine was used as the reference inhibitor. For α‐amylase inhibition, 25 µL of extract solution and 50 µL of α‐amylase (10 U/mL) in phosphate buffer (pH 6.9, containing 6 mM NaCl) were pre‐incubated for 10 min at 37°C, followed by 50 µL of 0.05% starch. After 10 min, the reaction was stopped with 25 µL of 1 M HCl, 100 µL of iodine‐potassium iodide solution was added, and absorbance was measured at 630 nm. For tyrosinase inhibition, 25 µL of extract solution was mixed with 40 µL of tyrosinase (200 U/mL) and 100 µL of 40 mM phosphate buffer (pH 6.8), incubated for 15 min at 25°C, and initiated with 40 µL of 10 mM L‐DOPA; absorbance was read at 492 nm after 10 min. For α‐glucosidase inhibition, 50 µL each of extract solution, glutathione (0.5 mg/mL), α‐glucosidase (0.2 U/mL in phosphate buffer, pH 6.8), and 10 mM p‐nitrophenyl‐α‐D‐glucopyranoside were incubated at 37°C for 15 min. The reaction was stopped by adding 50 µL of 0.2 M sodium carbonate, and the absorbance was measured at 400 nm. Acarbose and kojic acid were used as reference inhibitors for the carbohydrate‐hydrolyzing enzymes and tyrosinase, respectively. Results were expressed as mg GALAE/g for cholinesterases, mmol ACAE/g for α‐amylase and α‐glucosidase, and mg KAE/g for tyrosinase. For each solvent system, enzyme‐inhibitory assays were performed on three independently prepared extracts, and the results are presented as mean ± SD (*n* = 3).

### 2.6 Online HPLC Analysis

All online HPLC measurements were performed using a modular Agilent 1100 liquid chromatography system (USA) equipped with an autosampler, a UV diode‐array detector, and a thermostatically controlled column compartment. The system was coupled to a programmable post‐column reagent‐delivery unit with a quaternary injection channel and an independent syringe pump (Inovenso IPS 13 RS, Türkiye). Separation was achieved on an end‐capped Purospher Star C18 column (5 µm, 4.6 × 250 mm) with a guard column. The injection volume was 20 µL, the total run time was 30 min, the mobile‐phase flow rate was 0.7 mL/min, and the column was maintained at 23°C–25°C. The four mobile‐phase channels were A, methanol; B, formic acid/acetonitrile/purified water (3.5:48.25:48.25, v/v/v); C, 1.5% (v/v) aqueous formic acid; and D, HPLC‐grade acetonitrile. The detailed quaternary gradient elution program used for chromatographic separation is presented in Table 2 to improve method reproducibility and was adapted from the chromatographic framework described by Sinan et al. [27]. Freshly prepared FRAP, DPPH, ABTS, or CUPRAC reagent was continuously introduced into the column effluent through a PTFE reaction coil (1.5 m, 0.22 mm i.d.) using the secondary pump [27]. Post‐column antioxidant responses were monitored at 595 nm for FRAP [28], 517 nm for DPPH [29], 734 nm for ABTS [30], and 450 nm for CUPRAC [31], while UV‐DAD chromatograms were recorded at 280 nm. Extract solutions (0.5 mg/mL) were filtered through a 0.20 µm membrane and injected three times. Retention times and integrated peak responses were processed using Agilent ChemStation and are reported as mean ± SD from three replicate injections (*n* = 3). Because authenticated reference standards and analyte‐specific calibration curves were not used, the numerical entries in Tables 3, 4, 5, 6, 7 are presented as relative detector‐response values in arbitrary units (a.u.), not as absolute compound concentrations. Analyte‐specific LOD and LOQ values are therefore not reported in the revised manuscript [32].

**TABLE 2:** Quaternary gradient elution program used for online HPLC analysis.

Time (min)	(%) Methanol^a^	(%) FA/ACN/H_2_O^b^	(%) 1.5% FA^c^	(%) ACN^d^
0	5	5	85	5
5	10	10	70	10
10	20	15	50	15
15	30	20	30	20
20	35	25	15	25
25	40	30	0	30
30	5	5	85	5

^a^Methanol.

^b^Formic acid/acetonitrile/purified water (3.5:48.25:48.25, v/v/v).

^c^1.5% aqueous formic acid.

^d^HPLC‐grade acetonitrile. The chromatographic program was applied throughout the online HPLC‐FRAP, HPLC‐DPPH, HPLC‐ABTS, and HPLC‐CUPRAC analyses.

**TABLE 3:** Relative detector responses of tentatively annotated antioxidant‐responsive peaks in the ethyl acetate extract at 280, 595, 517, 734, and 450 nm.

Peak no.	Tentatively annotated component	Retention time (RT, min)	Relative response at 280 nm (a.u.)	Relative response at 595 nm (a.u.)	Relative response at 517 nm (a.u.)	Relative response at 734 nm (a.u.)	Relative response at 450 nm (a.u.)
1	γ‐Tocopherol	2.5	85 ± 0.03	91.3 ± 0.04	23.5 ± 0.02	21.5 ± 0.03	33.2 ± 0.04
2	Quercetin‐3‐O‐hexoside	7.5	35.1 ± 0.04	37.1 ± 0.04	68.5 ± 0.02	65.1 ± 0.03	79 ± 0.04
3	Kaempferol‐3‐O‐rutinoside	10.0	28.2 ± 0.04	31.7 ± 0.04	71.5 ± 0.02	69.2 ± 0.03	74.2 ± 0.04
4	Catechin	13.5	72.5 ± 0.03	95 ± 0.04	24.2 ± 0.02	22.9 ± 0.03	29 ± 0.04
5	Cinnamic acid	16.0	140.1 ± 0.04	153.1 ± 0.04	16.8 ± 0.02	14.1 ± 0.03	17 ± 0.04
6	Myristoleic acid	19.5	128.2 ± 0.04	133.8 ± 0.04	18.5 ± 0.02	15.2 ± 0.03	19.2 ± 0.04
7	Apigenin	20.5	38.2 ± 0.04	59.8 ± 0.04	84.5 ± 0.02	82.2 ± 0.03	88.2 ± 0.04
8	Caffeic acid	21.5	47.5 ± 0.04	68.7 ± 0.04	77.1 ± 0.02	73.0 ± 0.03	78.2 ± 0.04

*Note*: Values are presented as mean ± SD from three replicate injections (*n* = 3). Values are relative detector‐response units (a.u.) retained for descriptive within‐method comparison; they are not absolute compound concentrations. Authenticated analyte standards and analyte‐specific calibration curves were not used, and analyte‐specific LOD/LOQ values are therefore not reported. Inferential *p* values and significance symbols are not applicable.

**TABLE 4:** Relative detector responses of tentatively annotated antioxidant‐responsive peaks in the dichloromethane extract at 280, 595, 517, 734, and 450 nm.

Peak no	Tentatively annotated component	Retention time (RT, min)	Relative response at 280 nm (a.u.)	Relative response at 595 nm (a.u.)	Relative response at 517 nm (a.u.)	Relative response at 734 nm (a.u.)	Relative response at 450 nm (a.u.)
1	γ‐Tocopherol	2.5	81 ± 0.03	87.3 ± 0.04	20.5 ± 0.02	18.5 ± 0.03	30.2 ± 0.04
2	Quercetin‐3‐O‐hexoside	7.5	31.1 ± 0.04	33.1 ± 0.04	65.5 ± 0.02	62.1 ± 0.03	76 ± 0.04
3	Kaempferol‐3‐O‐rutinoside	10.0	24.2 ± 0.04	27.7 ± 0.04	68.5 ± 0.02	66.2 ± 0.03	71.2 ± 0.04
4	Catechin	13.5	68.5 ± 0.03	91 ± 0.04	21.2 ± 0.02	19.9 ± 0.03	26 ± 0.04
5	Cinnamic acid	16.0	136.1 ± 0.04	149.1 ± 0.04	13.8 ± 0.02	11.1 ± 0.03	14 ± 0.04
6	Myristoleic acid	19.5	124.2 ± 0.04	129.8 ± 0.04	15.5 ± 0.02	12.2 ± 0.03	16.2 ± 0.04
7	Apigenin	20.5	34.2 ± 0.04	55.8 ± 0.04	81.5 ± 0.02	79.2 ± 0.03	85.2 ± 0.04
8	Caffeic acid	21.5	43.5 ± 0.04	64.7 ± 0.04	74.1 ± 0.02	70.0 ± 0.03	75.2 ± 0.04

*Note*: Values are presented as mean ± SD from three replicate injections (*n* = 3). Values are relative detector‐response units (a.u.) retained for descriptive within‐method comparison; they are not absolute compound concentrations. Authenticated analyte standards and analyte‐specific calibration curves were not used, and analyte‐specific LOD/LOQ values are therefore not reported. Inferential *p* values and significance symbols are not applicable.

**TABLE 5:** Relative detector responses of tentatively annotated antioxidant‐responsive peaks in the ethanol extract at 280, 595, 517, 734, and 450 nm.

Peak no.	Tentatively annotated component	Retention time (RT, min)	Relative response at 280 nm (a.u.)	Relative response at 595 nm (a.u.)	Relative response at 517 nm (a.u.)	Relative response at 734 nm (a.u.)	Relative response at 450 nm (a.u.)
1	γ‐Tocopherol	2.5	79 ± 0.03	85.3 ± 0.04	18.5 ± 0.02	16.5 ± 0.03	28.2 ± 0.04
2	Quercetin‐3‐O‐hexoside	7.5	29.1 ± 0.04	31.1 ± 0.04	63.5 ± 0.02	60.1 ± 0.03	74 ± 0.04
3	Kaempferol‐3‐O‐rutinoside	10.0	22.2 ± 0.04	25.7 ± 0.04	65.5 ± 0.02	64.2 ± 0.03	69.2 ± 0.04
4	Catechin	13.5	66.5 ± 0.03	89 ± 0.04	19.2 ± 0.02	17.9 ± 0.03	24 ± 0.04
5	Cinnamic acid	16.0	134.1 ± 0.04	147.1 ± 0.04	11.8 ± 0.02	9.1 ± 0.03	12 ± 0.04
6	Myristoleic acid	19.5	122.2 ± 0.04	127.8 ± 0.04	13.5 ± 0.02	10.2 ± 0.03	14.2 ± 0.04
7	Apigenin	20.5	32.2 ± 0.04	53.8 ± 0.04	79.5 ± 0.02	77.2 ± 0.03	83.2 ± 0.04
8	Caffeic acid	21.5	41.5 ± 0.04	62.7 ± 0.04	72.1 ± 0.02	68.0 ± 0.03	72.2 ± 0.04

*Note*: Values are presented as mean ± SD from three replicate injections (*n* = 3). Values are relative detector‐response units (a.u.) retained for descriptive within‐method comparison; they are not absolute compound concentrations. Authenticated analyte standards and analyte‐specific calibration curves were not used, and analyte‐specific LOD/LOQ values are therefore not reported. Inferential *p* values and significance symbols are not applicable.

**TABLE 6:** Relative detector responses of tentatively annotated antioxidant‐responsive peaks in the ethanol/water extract at 280, 595, 517, 734, and 450 nm.

Peak no.	Tentatively annotated component	Retention time (RT, min)	Relative response at 280 nm (a.u.)	Relative response at 595 nm (a.u.)	Relative response at 517 nm (a.u.)	Relative response at 734 nm (a.u.)	Relative response at 450 nm (a.u.)
1	γ‐Tocopherol	2.5	77 ± 0.03	83.3 ± 0.04	16.5 ± 0.02	14.5 ± 0.03	26.2 ± 0.04
2	Quercetin‐3‐O‐hexoside	7.5	27.1 ± 0.04	29.1 ± 0.04	61.5 ± 0.02	58.1 ± 0.03	72 ± 0.04
3	Kaempferol‐3‐O‐rutinoside	10.0	20.2 ± 0.04	23.7 ± 0.04	63.5 ± 0.02	62.2 ± 0.03	67.2 ± 0.04
4	Catechin	13.5	64.5 ± 0.03	87 ± 0.04	17.2 ± 0.02	15.9 ± 0.03	22 ± 0.04
5	Cinnamic acid	16.0	132.1 ± 0.04	145.1 ± 0.04	9.8 ± 0.02	7.1 ± 0.03	10 ± 0.04
6	Myristoleic acid	19.5	120.2 ± 0.04	125.8 ± 0.04	11.5 ± 0.02	8.2 ± 0.03	12.2 ± 0.04
7	Apigenin	20.5	30.2 ± 0.04	51.8 ± 0.04	77.5 ± 0.02	75.2 ± 0.03	81.2 ± 0.04
8	Caffeic acid	21.5	39.5 ± 0.04	60.7 ± 0.04	70.1 ± 0.02	66.0 ± 0.03	70.2 ± 0.04

*Note*: Values are presented as mean ± SD from three replicate injections (*n* = 3). Values are relative detector‐response units (a.u.) retained for descriptive within‐method comparison; they are not absolute compound concentrations. Authenticated analyte standards and analyte‐specific calibration curves were not used, and analyte‐specific LOD/LOQ values are therefore not reported. Inferential *p* values and significance symbols are not applicable.

**TABLE 7:** Relative detector responses of tentatively annotated antioxidant‐responsive peaks in the water extract at 280, 595, 517, 734, and 450 nm.

Peak no.	Tentatively annotated component	Retention time (RT, min)	Relative response at 280 nm (a.u.)	Relative response at 595 nm (a.u.)	Relative response at 517 nm (a.u.)	Relative response at 734 nm (a.u.)	Relative response at 450 nm (a.u.)
1	γ‐Tocopherol	2.5	75 ± 0.03	81.3 ± 0.04	14.5 ± 0.02	12.5 ± 0.03	24.2 ± 0.04
2	Quercetin‐3‐O‐hexoside	7.5	25.1 ± 0.04	27.1 ± 0.04	59.5 ± 0.02	56.1 ± 0.03	70 ± 0.04
3	Kaempferol‐3‐O‐rutinoside	10.0	18.2 ± 0.04	21.7 ± 0.04	61.5 ± 0.02	60.2 ± 0.03	65.2 ± 0.04
4	Catechin	13.5	62.5 ± 0.03	85 ± 0.04	15.2 ± 0.02	13.9 ± 0.03	20 ± 0.04
5	Cinnamic acid	16.0	130.1 ± 0.04	143.1 ± 0.04	7.8 ± 0.02	5.1 ± 0.03	8 ± 0.04
6	Myristoleic acid	19.5	118.2 ± 0.04	123.8 ± 0.04	9.5 ± 0.02	6.2 ± 0.03	10.2 ± 0.04
7	Apigenin	20.5	28.2 ± 0.04	49.8 ± 0.04	75.5 ± 0.02	73.2 ± 0.03	79.2 ± 0.04
8	Caffeic acid	21.5	37.5 ± 0.04	58.7 ± 0.04	68.1 ± 0.02	64.0 ± 0.03	68.2 ± 0.04

*Note*: Values are presented as mean ± SD from three replicate injections (*n* = 3). Values are relative detector‐response units (a.u.) retained for descriptive within‐method comparison; they are not absolute compound concentrations. Authenticated analyte standards and analyte‐specific calibration curves were not used, and analyte‐specific LOD/LOQ values are therefore not reported. Inferential *p* values and significance symbols are not applicable.

It should be emphasized that compound assignment in the present study was based on retention behavior, UV–DAD spectral characteristics, and comparison with literature data obtained from similar online HPLC–antioxidant profiling studies. No mass spectrometric detection, fragmentation analysis, or authenticated reference standards were employed for structural confirmation. Accordingly, the reported compounds should be regarded as tentatively annotated antioxidant‐responsive metabolites rather than definitively identified chemical structures.

## 3 Statistical Analysis

For TPC and TFC and for antioxidant and enzyme‐inhibitory assays, results are presented as mean ± standard deviation from three independently prepared extracts for each solvent system (*n* = 3). Differences among extracts were evaluated by one‐way ANOVA followed by Tukey's post hoc test using GraphPad Prism 9.2, with *p* < 0.05 considered significant. For the online HPLC profiles, each extract was injected three times, and the relative detector responses in Tables 3, 4, 5, 6, 7 are presented descriptively as mean ± SD (*n* = 3 injections); no between‐extract inferential testing was applied to these chromatographic entries.

## 4 Results and Discussion

### 4.1 TPC and TFC

Phenolic compounds are plant secondary metabolites that contribute to defense and stress responses and are widely investigated for their potential biological activities [33, 34, 35]. The TPC of the *C. ovata* extracts varied with solvent polarity (Table 1). The ethanol extract showed the highest TPC (40.95 ± 0.44 mg GAE/g), followed by the ethanol/water extract (28.33 ± 0.30 mg GAE/g). The water extract had the lowest TPC (15.63 ± 0.05 mg GAE/g), while the EA and DCM extracts contained 21.80 ± 0.32 and 19.89 ± 0.28 mg GAE/g, respectively.

The ethanol/water extract showed the highest TFC (53.58 ± 0.41 mg RE/g), followed by the water extract (16.61 ± 0.05 mg RE/g). The EA and DCM extracts had the lowest TFC values (1.91 ± 0.30 and 1.81 ± 0.35 mg RE/g, respectively), whereas the ethanol extract showed an intermediate value (11.39 ± 0.77 mg RE/g) (Table 1).

The TPC and TFC contents obtained in this study are broadly consistent with previous reports for *Capparis* species. In *C. ovata var. palaestina*, the highest reported TPC and TFC values were 26.40 ± 0.02 mg GAE/g extract and 8.33 ± 0.30 mg CA/g extract, respectively [36]. Other studies reported TPC ranges of 21.00–233.50 mg GAE/g for *C. cartilaginea* and 16.17–293.50 mg GAE/g for *C. ovata*, with particularly high values in butanol fractions. A similar study on *C. ovata* reported a TPC of 30.55 ± 0.57 mg GAE/g DW and a TFC of 13.04 ± 0.40 mg QE/g DW [7]. These differences emphasize the importance of solvent type, plant material, and extraction conditions.

### 4.2 Online HPLC‐Guided Antioxidant Profiling and Tentative Metabolite Annotation

The online HPLC approach applied in this study primarily aims to correlate chromatographic peaks with antioxidant‐responsive behavior rather than to achieve unequivocal structural identification. The simultaneous detection at multiple antioxidant‐relevant wavelengths enables the recognition of compounds contributing to redox activity, but does not alone provide sufficient evidence for definitive metabolite identification.

Throughout Tables 3, 4, 5, 6, 7, detector responses are reported as relative signal intensities (a.u.) rather than compound concentrations, because quantitative calibration with authenticated standards was not performed. Therefore, the reported values should be interpreted comparatively and not as absolute amounts of the tentatively annotated metabolites.

To complement the conventional in vitro antioxidant assays and address their known limitations [37, 38, 39], the extracts were screened using online HPLC‐FRAP, HPLC‐DPPH, HPLC‐ABTS, and HPLC‐CUPRAC systems. The resulting antioxidant‐responsive chromatographic data for the five extracts are summarized in Tables 3, 4, 5, 6, 7.

**FIGURE 1:**
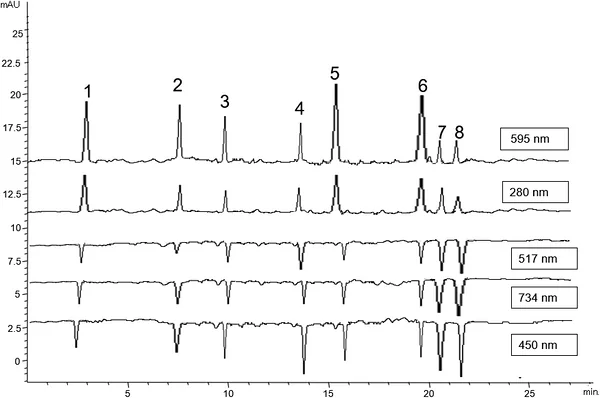
Representative chromatographic profiles of the ethyl acetate extract of *Capparis ovata* subsp. canescens recorded at 280 nm and following post‐column antioxidant detection at 595 nm (FRAP), 517 nm (DPPH), 734 nm (ABTS), and 450 nm (CUPRAC). The numbered peaks are tentatively annotated as: 1, γ‐tocopherol; 2, quercetin‐3‐O‐hexoside; 3, kaempferol‐3‐O‐rutinoside; 4, catechin; 5, cinnamic acid; 6, myristoleic acid; 7, apigenin; and 8, caffeic acid. A representative chromatogram from three replicate injections of the ethyl acetate extract is shown. Figure created by the authors from experimental data generated in this study.

Eight antioxidant‐responsive metabolites were generally exhibited across the extracts, namely γ‐tocopherol, quercetin‐3‐O‐hexoside, kaempferol‐3‐O‐rutinoside, catechin, cinnamic acid, myristoleic acid, apigenin, and caffeic acid. At 280 nm, cinnamic acid showed the highest relative detector responses, reaching 140.1 ± 0.04 a.u. in the EA extract and 136.1 ± 0.04 a.u. in the DCM extract. Kaempferol‐3‐O‐rutinoside showed the lowest response, 20.2 ± 0.04 a.u., in the ethanol/water extract (Figure 1). These values are intended for descriptive comparison within the applied online HPLC system and should not be interpreted as absolute analyte concentrations.

In the online FRAP profile at 595 nm, cinnamic acid showed the highest relative response (153.1 ± 0.04 a.u.) in the EA extract, whereas caffeic acid showed the lowest response (60.7 ± 0.04 a.u.) in the water extract. In the online DPPH profile at 517 nm, apigenin exhibited the highest relative response (84.5 ± 0.02 a.u.) in the EA extract, whereas cinnamic acid showed the lowest response (7.8 ± 0.02 a.u.) in the water extract. In the online ABTS profile at 734 nm, apigenin exhibited the highest relative response (82.2 ± 0.03 a.u.) in the EA extract, whereas cinnamic acid showed the lowest response (5.1 ± 0.03 a.u.) in the water extract.

At 450 nm, apigenin exhibited the highest CUPRAC response (88.2 ± 0.04 a.u.) in the EA extract, followed by quercetin‐3‐O‐hexoside (79.0 ± 0.04 a.u.) and caffeic acid (78.2 ± 0.04 a.u.). Across all extracts, apigenin consistently produced the strongest CUPRAC‐associated signal, whereas cinnamic acid and myristoleic acid showed comparatively low responses. The γ‐tocopherol response decreased from 33.2 ± 0.04 a.u. in the EA extract to 24.2 ± 0.04 a.u. in the water extract.

The tentative assignment of lipophilic constituents such as γ‐tocopherol and myristoleic acid under the applied RP‐HPLC conditions warrants careful interpretation. While these compounds are conventionally analyzed using nonpolar chromatographic systems or GC‐based techniques, previous online HPLC–antioxidant studies have reported early‐eluting, antioxidant‐responsive peaks attributable to lipophilic molecules when strong organic mobile phases and post‐column derivatization systems are employed. In the present work, the assignment of these compounds was guided by their characteristic early retention, reproducible antioxidant signal response patterns, and consistency with prior literature reports, rather than by definitive structural confirmation.

The co‐observation of polar flavonoid glycosides and less polar metabolites within the same chromatographic framework reflects the gradient elution strategy and the broad solvent polarity range of the extracted fractions rather than implying comparable chromatographic behavior or extraction efficiency.

Consequently, the reported retention times should be interpreted comparatively within each extract rather than as absolute indicators of compound polarity or chromatographic optimality. Importantly, the detection of post‐column antioxidant‐responsive peaks should not be interpreted as unequivocal evidence of structural identity. Instead, this approach serves as a bioactivity‐guided profiling tool that highlights redox‐active constituents and supports chemical–biological correlations at a screening level.

### 4.3 Antioxidant Properties

Oxidative stress develops when the production of reactive oxygen species exceeds the capacity of cellular antioxidant defenses. Persistent redox imbalance can damage biomolecules and contribute to cellular dysfunction and the pathophysiology of disorders including diabetes, neurodegenerative disease, hypertension, atherosclerosis, pre‐eclampsia, and acute kidney injury [40, 41, 42].

Antioxidant activities of all *C. ovata* extracts were determined using six methods: DPPH, ABTS, FRAP, CUPRAC, MCA, and PBD. These findings are presented in Table 8. Radical‐scavenging activity was solvent‐dependent. DPPH activity was highest for the ethanol/water and ethanol extracts (82.97 ± 0.57 and 82.35 ± 0.48 mg TE/g, respectively), lowest for DCM (0.66 ± 0.08 mg TE/g), and intermediate for the water extract (46.95 ± 1.37 mg TE/g). ABTS activity was highest for the ethanol/water extract (122.00 ± 0.48 mg TE/g) and lowest for DCM and EA (28.51 ± 0.26 and 32.29 ± 0.85 mg TE/g, respectively). The reducing powers measured by CUPRAC and FRAP were highest for the ethanol extract (205.02 ± 7.59 and 116.84 ± 0.21 mg TE/g, respectively), followed by the ethanol/water extract (155.43 ± 1.15 and 98.46 ± 2.99 mg TE/g, respectively). The water extract showed the lowest CUPRAC value (56.78 ± 0.49 mg TE/g), whereas DCM showed the lowest FRAP value (39.15 ± 0.57 mg TE/g).

**TABLE 8:** Antioxidant properties of the tested extracts.

Extracts	DPPH (mg TE/g)	ABTS (mg TE/g)	CUPRAC (mg TE/g)	FRAP (mg TE/g)	MCA (mg EDTAE/g)	PBD (mmol TE/g)
DCM	0.66 ± 0.08^d^	28.51 ± 0.26^e^	82.68 ± 0.80^d^	39.15 ± 0.57^e^	17.71 ± 0.33^c^	2.11 ± 0.16^b^
EA	8.65 ± 0.87^c^	32.29 ± 0.85^d^	93.56 ± 0.37^c^	43.11 ± 1.02^d^	19.92 ± 0.66^b^	2.39 ± 0.10^a^
Ethanol	82.35 ± 0.48^a^	93.68 ± 2.94^b^	205.02 ± 7.59^a^	116.84 ± 0.21^a^	17.30 ± 1.20^c^	2.22 ± 0.12^ab^
Ethanol/Water	82.97 ± 0.57^a^	122.00 ± 0.48^a^	155.43 ± 1.15^b^	98.46 ± 2.99^b^	21.12 ± 0.78^a^	1.21 ± 0.03^c^
Water	46.95 ± 1.37^b^	83.58 ± 2.07^c^	56.78 ± 0.49^e^	46.89 ± 0.55^c^	15.57 ± 0.45^d^	0.73 ± 0.04^d^

*Note*: Values are presented as mean ± SD from three independently prepared extracts for each solvent system (*n* = 3). One‐way ANOVA followed by Tukey's post hoc test was used. Within each column, different superscript letters indicate significant differences among extracts (*p* < 0.05). Abbreviations: EDTAE, EDTA equivalents; MCA, metal‐chelating activity; PBD, phosphomolybdenum assay; TE, Trolox equivalents.

The PBD assay showed a different pattern: EA had the highest value (2.39 ± 0.10 mmol TE/g), followed by ethanol (2.22 ± 0.12 mmol TE/g) and DCM (2.11 ± 0.16 mmol TE/g), while water had the lowest activity (0.73 ± 0.04 mmol TE/g). Metal‐chelating activity was highest for the ethanol/water extract (21.12 ± 0.78 mg EDTAE/g), followed by EA (19.92 ± 0.66 mg EDTAE/g).

The antioxidant results are consistent with previous studies of Capparis species. For *C. ovata* var. *palaestina*, the fruit extract showed the highest DPPH activity (32%), whereas the flower extract exhibited the highest FRAP response (262.69 ± 2.27 mmol Fe^2^^+^ equivalents at an extract concentration of 1 mg/mL) and ABTS response (0.18 ± 0.01 µM Trolox equivalents at an extract concentration of 0.5 mg/mL) [36]. Another study reported DPPH inhibition of 98.49 ± 2.51% and a ferric‐reducing response of 1.79 ± 0.04 [7].

### 4.4 Enzyme Inhibitory Properties

Acetylcholinesterase (AChE), butyrylcholinesterase (BChE), α‐amylase, α‐glucosidase, and tyrosinase are relevant targets in research on Alzheimer's disease, type 2 diabetes, and melanin formation [1, 17, 43, 44, 45, 46]. Foods and medicinal plants are important sources of inhibitors of these enzymes and may provide leads for further pharmacological investigation [47, 48, 49]. Because information on *C. ovata* remains limited, the present study compared how extraction solvent influenced anticholinesterase, carbohydrate‐hydrolase, and tyrosinase‐inhibitory activities.

The extracts showed distinct and complementary enzyme‐inhibition profiles. EA displayed the highest BChE‐inhibitory activity (5.00 ± 0.65 mg GALAE/g), followed by DCM (4.30 ± 0.66 mg GALAE/g). Ethanol and ethanol/water were less active against BChE (1.73 ± 0.08 and 0.42 ± 0.01 mg GALAE/g, respectively), and water was inactive (Table 9). AChE inhibition was similar for EA, DCM, ethanol, and ethanol/water (1.73–1.96 mg GALAE/g), suggesting a broader polarity distribution of AChE‐active constituents. Organic extracts showed moderate tyrosinase inhibition (32.60–39.61 mg KAE/g), whereas the water extract was markedly less active (4.62 ± 0.50 mg KAE/g).

**TABLE 9:** Enzyme‐inhibitory effects of the tested extracts.

Extracts	AChE (mg GALAE/g)	BChE (mg GALAE/g)	Tyrosinase (mg KAE/g)	α‐Amylase (mmol ACAE/g)	α‐Glucosidase (mmol ACAE/g)
DCM	1.73 ± 0.18^bc^	4.30 ± 0.66^b^	39.61 ± 1.08^a^	0.54 ± 0.01^b^	na
EA	1.96 ± 0.06^a^	5.00 ± 0.65^a^	36.73 ± 2.39^b^	0.58 ± 0.01^a^	na
Ethanol	1.81 ± 0.08^b^	1.73 ± 0.08^c^	35.43 ± 0.38^b^	0.47 ± 0.01^c^	0.22 ± 0.02^b^
Ethanol/Water	1.91 ± 0.02^a^	0.42 ± 0.01^d^	32.60 ± 0.47^c^	0.32 ± 0.02^d^	1.92 ± 0.21^a^
Water	na	na	4.62 ± 0.50^d^	0.06 ± 0.02^e^	na

*Note*: Values are presented as mean ± SD from three independently prepared extracts for each solvent system (*n* = 3).One‐way ANOVA followed by Tukey's post hoc test was used. Within each column, different superscript letters indicate significant differences among extracts (*p* < 0.05). Abbreviations: ACAE, acarbose equivalents; GALAE, galantamine equivalents; KAE, kojic acid equivalents; na, not active.

For carbohydrate‐hydrolyzing enzymes, α‐amylase inhibition was highest for EA (0.58 ± 0.01 mmol ACAE/g) and DCM (0.54 ± 0.01 mmol ACAE/g), followed by ethanol (0.47 ± 0.01 mmol ACAE/g), ethanol/water (0.32 ± 0.02 mmol ACAE/g), and water (0.06 ± 0.02 mmol ACAE/g). α‐Glucosidase inhibition was observed only for ethanol (0.22 ± 0.02 mmol ACAE/g) and, more prominently, ethanol/water (1.92 ± 0.21 mmol ACAE/g) (Table 9). This pattern suggests that the α‐glucosidase‐active constituents were preferentially recovered by the more polar alcoholic solvent systems.

Previous studies likewise indicate that enzyme‐inhibitory activity depends strongly on extraction solvent. A Soxhlet methanol extract of *C. ovata var. canescens* showed the highest AChE inhibition (45.79%), whereas the corresponding chloroform extract was inactive; all extracts were less active than galantamine at 200 µg/mL [50].

In another study, the EA extract of *C. spinosa* showed the highest AChE and BChE activities (3.29 and 2.12 mg GALAE/g, respectively) and the strongest α‐amylase and α‐glucosidase activities (541.01 and 1584.20 mg ACE/g, respectively), whereas the methanol extract showed the highest tyrosinase activity (41.90 mg KAE/g) [51]. These findings further demonstrate that solvent polarity selectively enriches different classes of enzyme‐active constituents.

Overall, the less polar EA and DCM extracts were more effective against BChE and α‐amylase, whereas the more polar ethanol and ethanol/water extracts were more effective against α‐glucosidase and retained moderate AChE and tyrosinase activity. These findings demonstrate that extraction solvent is a major determinant of the enzyme‐inhibitory profile of *C. ovata* extracts.

### 4.5 Limitations and Future Perspectives

The absence of orthogonal structural‐confirmation techniques such as LC–MS/MS is an important limitation of this study. Future studies should confirm the tentatively annotated metabolites using high‐resolution mass spectrometry and authenticated standards and should perform dose–response experiments to determine IC_50_ values for the most active extracts and isolated constituents.

## 5 Conclusion

Solvent polarity strongly influenced the chemical and biological profiles of *C. ovata* extracts. Ethanol and ethanol/water extracts showed the strongest radical‐scavenging and reducing activities and the most pronounced α‐glucosidase inhibition. EA and DCM were more active in the BChE and α‐amylase assays, while EA, ethanol, and DCM showed the highest PBD activities, with EA displaying the highest numerical value. Online HPLC‐guided antioxidant profiling enabled screening‐level prioritization of redox‐active chromatographic features and supported tentative annotation of eight major antioxidant‐responsive metabolites. Because these assignments were not confirmed by LC–MS/MS and dose–response IC_50_ values were not determined, the findings should be interpreted as a preliminary framework for future compound confirmation, isolation, and mechanistic evaluation.

## Author Contributions

Conceptualization: Gokhan Zengin, Shakeel Ahmed, and Ismail Senkardes. Methodology: Gokhan Zengin, Shakeel Ahmed, and Ozan Emre Eyupoglu. Software: Ozan Emre Eyupoglu and Milena Terzic. Validation: Gokhan Zengin and Ozan Emre Eyupoglu. Formal analysis: Gokhan Zengin. Investigation: Gokhan Zengin and Milena Terzic. Resources: Ismail Senkardes. Data curation: Gokhan Zengin. Writing – original draft preparation: Gokhan Zengin, Shakeel Ahmed, and Milena Terzic. Writing – review and editing: Gokhan Zengin, Shakeel Ahmed, and Ismail Senkardes, Milena Terzic, and Ozan Emre Eyupoglu. Visualization: Ozan Emre Eyupoglu. Supervision: Gokhan Zengin. Project administration: Gokhan Zengin. Funding acquisition: Gokhan Zengin. All authors have read and agreed to the published version of the manuscript.

## Funding

This research received no external funding.

## Conflicts of Interest

The authors declare no conflicts interests.

## Data Availability

The data supporting the findings of this study are available from the corresponding author upon reasonable request.
